# Identification of a mature cystic teratoma mimicking a presacral tumor by transsacral surgery in a young female: A case report

**DOI:** 10.3892/ol.2013.1453

**Published:** 2013-07-09

**Authors:** YI YANG, XIAOXIAO WANG, ZHENYANG LI, JIANBIN XIANG, ZONGYOU CHEN

**Affiliations:** Department of General Surgery, Huashan Hospital, Fudan University, Shanghai 200040, P.R. China

**Keywords:** mature cystic teratoma, ovary, presacral tumor, transsacral approach

## Abstract

The current case report presents an account of a unique surgical procedure performed to remove an extremely rare occurrence of a transsacral abdominal mass from a 24-year-old female. The patient presented with subtle sacrococcygeal pain for two months and a presacral abdominal mass derived from the right ovary. The mass was misdiagnosed as a presacral tumor based on the results of magnetic resonance imaging (MRI) performed prior to the surgery. The patient also exhibited the symptoms commonly caused by a presacral mass, however, during the surgery, the mass was not initially located under the sacrum. An ultrasound examination and an analysis of an intraoperative frozen section indicated that the mass was a mature cystic teratoma (MCT) of the ovary, located in the peritoneal cavity between the rectum and uterus. The mass was successfully resected and removed from the affected ovary through the abdominal cavity via the sacral region. A pathological examination of the tumor section confirmed a diagnosis of a MCT of the ovary.

## Introduction

Mature cystic teratomas (MCTs) are germ cell tumors that commonly occur in the ovaries, accounting for 10% of all ovarian tumors (1). Ovarian MCTs are usually located in the uterine adnexa and pouch of Douglas, where the tumors arise from the nervous system (i.e. chordoma and schwannoma) or from the intestinal tract (i.e. colorectal carcinoma and mesenchymoma) in the presacral region. Teratomas, particularly ovarian MCTs, rarely occur at presacral locations (2). Transsacral surgery is a routine procedure for presacral tumor management. The current study presents an extremely rare case of transsacral abdominal ovarian MCT and describes the surgical removal of this tumor.

## Case report

### Clinical presentation and diagnosis

**Figure 1:**
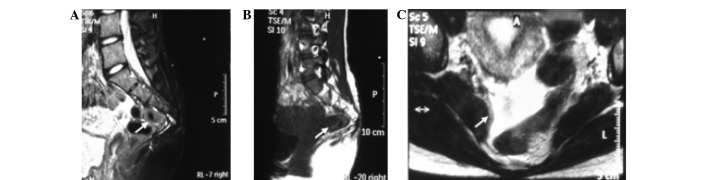
Magnetic resonance imaging (MRI) of the pelvis. (A) The mass (arrow) was detected under the sacrum (T1WI). (B) The mass (arrow) in a T2WI. (C) The mass at the right side of the rectum and with fat-like and soft tissue components. WI, weighted image.

**Figure 2:**
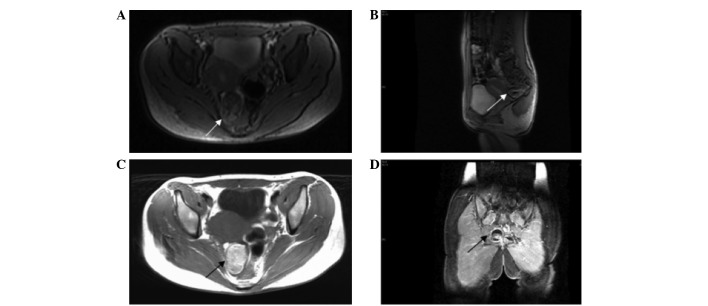
Enhanced magnetic resonance imaging (MRI) of the pelvis. (A) A transverse section of the mass (arrow) in a T1WI. (B) A sagital section of the mass (arrow) in a T1WI, posterior to the uterus and approaching the sacrum. (C) A transverse section of the mass (arrow) in aT2WI. (D) A coronal section of the mass (arrow) revealed that the mass was located in the right region of the pelvic cavity, at the right side of the rectum.

A 24-year-old Mongolian female was referred to the outpatient clinic at Huashan Hospital (Shanghai, China) with mild sacrococcygeal pain that had been present for two months with no obvious causation. The individual had no fever, abdominal pain or changes in habitual normal bowel movement and urination. Magnetic resonance imaging (MRI) of the patient’s pelvis revealed a 53×40×33-mm pelvic mass, posterior to the sacrum and at the right side of the rectum (Fig. 1). An enhanced MRI showed that the mass, a possible teratoma, was under the sacrum (Fig. 2). Based on the radiological features, the mass was located at the posterior of the rectum towards the right side and was extended near the sacrum with clear boundaries without evidence of sacral invasion. A laboratory examination of the tumor section, including an analysis for tumor markers CA 19-9 and CA 125, revealed results within the normal ranges. The patient’s medical history included a coccyx fracture in 2002 and a sacral epidermal cystectomy in 2004.

Upon admission to the hospital, no abdominal tenderness, rebound pain or palpable masses were identified by a physical examination. The initial rectal exam detected the inferior end of a non-mobile, non-tender and smooth-edged mass attached to the right posterior side of the rectum.

### Surgical procedure

**Figure 3:**
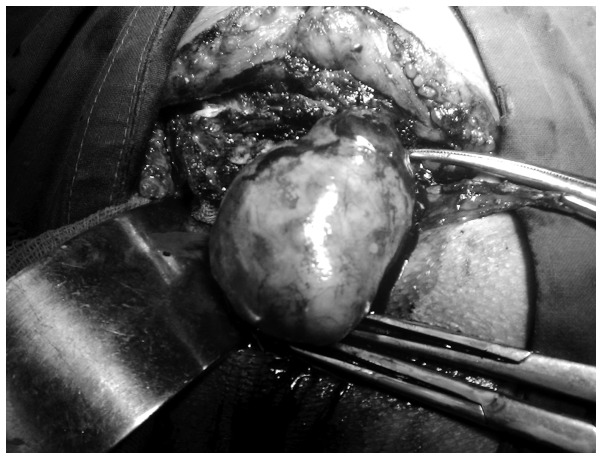
Intraoperative image. The mass was rezected out of the abdominal cavity from the sacral region without difficulty. The mass was encapsulated by normal ovarian-like tissue.

The patient was scheduled for surgical treatment under general anesthesia two days after admission. Intraoperatively, the patient was placed in the prone jack-knife position. The surgery was performed with a 10-cm incision at the inferior margin of the fifth sacrum. The coccyx and the inferior margin of the fifth sacrum were removed. The right side of the wall of the rectum was dissociated from the presacral rectum by an incision into the gorge of the sacrum. No tumor mass was located by laparotomy or the initial examination during surgery. Next, an ultrasound examination was performed and the mass was visualized at the right side of rectum, posterior to the uterus. The right ovary was not located at its normal anatomical site. The peritoneum was opened and the enlarged right ovary was identified as a presacral mass with an irregular appearance. The mass had a diameter of 5 cm and its outer boundary was smooth with no feeding vessel or ligamentous connection to the surrounding organs, including the rectum, uterus, cervix and vagina. The sacrum and coccyx were unaffected (Fig. 3). The left ovary was normal in appearance and located at its usual anatomical site.

The right ovary tumor mass was resected and removed from the normal ovarian tissue by a gynecologist. The examination of an intraoperative frozen section indicated a possible diagnosis of a presacral MCT (dermoid cyst). A further pathological examination confirmed the diagnosis of a presacral MCT. The patient recovered uneventfully and was discharged 6 days after the surgery. Written informed consent was obtained from the patient for the publication of this case report.

## Discussion

Teratomas arise mainly from totipotential gonadal cells, and while a number of them may have malignant potential, the majority are benign at the time of identification. The cause of teratomas remains unknown (3). These masses are encapsulated tumors with components that resemble normal derivatives of all three germ layers and they appear completely different to the normal tissue in which they are embedded. Teratomas represent 10–15% of the total number of recorded ovarian tumors and are commonly found in young patients (4). MCTs are the most common type of teratoma, accounting for the majority of germ cell tumors. These tumors generally develop as a single mass away from the midline (5). The symptoms of teratomas vary between individuals, but these tumors are mainly identified as a result of organ occupation and compression by the mass. Presacral masses arise from the nervous system or intestinal tract. In contrast to the current case report, primary presacral MCTs are one of the most common types of tumor to be found in infants and are rarely reported in adults (6). Primary presacral MCTs occur more frequently in females, and the female-to-male ratio is 10:1 (7).

An accurate diagnosis of a presacral mass prior to surgical removal is extremely important. A number of standard diagnostic imaging methods, including ultrasonography (transvaginal and transrectal), computed tomography (CT) and MRI, are readily available and must be used to generate a detailed image of the mass to facilitate pre-operative planning (8,9). Although presacral ovarian MCTs are extremely rare, to avoid any diagnostic dilemma, a gynecological ultrasound examination via the vagina and/or rectum is an important routine examination. Sonographically, an MCT may present predominantly as a cyst, containing a solid or complex tumor mass (10). Gynecological ultrasound examinations are able to reveal the anatomical location of the tumor and its relationship to the adnexa uteri. This technique is the most commonly employed imaging method for the assessment of pathological conditions associated with the adnexa uteri. Regardless of whether transsacral or transabdominal surgery is performed, an ultrasound may be useful for monitoring and adjusting the surgical approach.

Radiological visualization techniques, including CT and MRI, enable us to generate a pre-operative diagnosis for presacral tumors (11,12). CT scans are useful for determining the size and density of the lesion and its spatial relations, however, they lack the specificity to differentiate the types of presacral tumors (13). MCTs typically show various CT-based attenuations. The considerably variable MRI signal intensities generated by the various tissue contents of the mass within the cystic lesion, including fat and bone, are characteristic of MCTs. MRI has also been used to evaluate presacral tumors and is known to be able to define the type of tumor as well as visualize and screen for meningeal and bone invasion (14).

In the current patient, MRI showed the fat-like and soft tissue components within the presacral mass. Since a diagnosis of a primary presacral MCT could not be ruled out, particularly due to the patient’s medical history and physical examination results, the transsacral approach was selected for the surgery.

Surgical resection is the standard curative treatment for the majority of MCTs. For patients who are otherwise healthy and whose lesions appear resectable, the management of such lesions is almost always surgical resection. The approach depends on the nature and location of the lesion. Previous studies have reported that lesions which do not extend below S4 should be resected transabdominally, while lesions below S4 and <8 cm in diameter should be resected transsacrally and larger lesions or those in an intermediate position may require a combined abdominal and sacral surgical procedure (15,16). According to the MRI results in the present study, the lesion of the patient was >5 cm in diameter and below S5. Therefore, the transsacral approach was selected to access and remove the tumor.

The final diagnosis for this patient was a transsacral ovarian MCT in the presacral region. Ovarian MCTs mostly occur in the pelvic cavity and are commonly observed in the pouch of Douglas and occasionally in the uterosacral ligament (17). In rare cases, masses have been identified in the presacral region (18,19). The location of a mass in the presacral region is likely to be due to gravitational effects on the relatively large mass size, the heterogeneous content of the mass that enables it to be squeezed and reshaped along with the surroundings during exogenic movements or compressions and the presence of a smooth outer layer with limited attachments, together with the heterotypic or displaced of the ovary. In the current case, the MCT had a diameter of 5 cm and exhibited heterogeneous density. The mass was tucked into the right inferior of the sacrum, which may have been due to gravitational pull and the compression force of bowel movements. Several radiological examinations did show that the tumor was at this location. We hypothesized that the supine position assumed during the examination may have also contributed to such a result; the mass was eventually located in the position it had descended to in the presacral region. These effects contributed to the prediction of the derivation and location of the tumor.

In addition, the most frequent symptom of MCT is lower abdominal pain (20), while the most common symptoms of patients with presacral masses are suppuration and sacrococcygeal pain (21). The current patient reported symptoms similar to those caused by a presacral mass, an additional contribution to the misdiagnosis.

Performing a laparotomy with an uncertain target and an unplanned surgical approach must be prevented to limit surgery-related trauma and complications. During transsacral surgery to remove a presacral mass, ultrasonography must be performed if the mass cannot be located initially.

In the present case, no mass was located by the laparotomy or initial examination during the surgery. An ultrasound examination was performed, confirming that the mass was at the right side of rectum, posterior to the uterus and embedded in the predicted right ovary, which was not at its normal anatomical location. Since the tumor was covered by normal smooth ovarian tissue and had no adhesion to other organs and as the patient was in the prone jack-knife position, the likelihood that the tumor may have moved and descended into the abdominal cavity must be considered.

We resected and dissociated the tumor mass from the ovary through the sacral region and returned the ovary to the normal anatomical location. The organs posterior to the bladder were exposed in a clear view to avoid injuring the ureters. As MCTs are curable in the majority of cases by a complete surgical resection of the tumor alone (22), and as the surgery is also sonographically monitored, operating through the sacral region is likely to have no negative impact on the outcome and prognosis of this case.

Ovarian MCTs commonly occur in the pelvic cavity and are rarely identified in the presacral position. In cases where ultrasound and radiological examinations strongly indicate the presence of a teratoma, a diagnosis of a prescacral ovarian MCT must be taken into consideration.

Pre-operative ultrasound and radiographic examinations must be performed in the supine and prone positions to assess the firmness and mobility of the presacral mass, as well as its derivation and relationship to the surroundings. Based on these efforts, the most efficient and least trauma-inflicting surgical approach must be determined prior to surgery.

To dissect a transsacral abdominal mass, an ultrasound examination must be performed during the surgery to assist in the identification of the mass if it is not firmly attached, movable or is not able to be located directly during surgery initially. If the transsacral laparotomy fails to locate the mass, an additional surgical procedure, such as a transparietal laparotomy, should be performed. To assess a transsacral mass, it is feasible that, during transsacral abdominal surgery, the peritoneum is opened from the pelvic floor. By contrast, for a common MCT that may be curatively managed by complete surgical resection transabdomenally, the transsacral approach is unnecessary and may be relatively more traumatic.

## References

[1] Peterson WF (1957). Malignant degeneration of benign cystic teratomas of the ovary; a collective review of the literature. Obstet Gynecol Surv.

[2] Heller DS, Keohane M, Bessim S, Jagirdar J, Deligdisch L (1989). Pituitary-containing benign cystic teratoma arising from the terosacral ligament. Arch Pathol Lab Med.

[3] Jucá M, de Oliveira FF, Gomes EG, Le Campion E (2006). Sacrococcygeal teratoma in adult. Report of a case. Int J Gastrointest Canc.

[4] Jeffcoate N, Tindal VR (1987). Jeffcoate’s Principles of Gynaecology.

[5] Krnojelac D, Hadzić B, Curcin N, Dolai M, Bogdanović G (1999). Malignant transformation of thyroid tissue in an ovarian dermoid cyst: case report. Med Pregl.

[6] Moawad NS, Starks D, Ashby K (2008). Ectopic ovarian teratoma of the uterosacral ligament associated with a large ovarian dermoid. J Minim Invasive Gynecol.

[7] Al-Essa AA, Malik TA, Baghdadi MK, El Tayeb AA (2004). Adult sacrococcygeal teratomas. Saudi Med J.

[8] Pidala MJ, Eisenstat TE, Rubin Rj, Salvati EP (1999). Presacral cysts: transrectal excision in select patients. Am Surg.

[9] Negro F, Mercuri M, Ricciardi V (2005). Presacral epidermoid cyst: a case report. Ann Ital Chir.

[10] Saba L, Guerriero S, Sulcis R, Virgilio B, Melis G, Mallarini G (2009). Mature and immature ovarian teratomas: CT, US and MR imaging characteristics. Eur J Radiol.

[11] Davidson AJ, Hartman DS, Goldman SM (1989). Mature teratoma of the retroperitoneum: radiologic, pathologic, and clinical correlation. Radiology.

[12] Pereira JM, Sirlin CB, Pinto PS, Casola G (2005). CT and MR imaging of extrahepatic fatty masses of the abdomen and pelvis: techniques, diagnosis, differential diagnosis, and pitfalls. Radiographics.

[13] Yang DM, Yoon MH, Kim HS (2001). Presacral epidermoid cyst: imaging findings with histopathologic correlation. Abdom Imaging.

[14] Riojas CM, Hahn CD, Johnson EK (2010). Presacral epidermoid cyst in a male: a case report and literature review. J Surg Educ.

[15] Bullard Dunn K (2010). Retrorectal tumors. Surg Clin North Am.

[16] Du F, Jin K, Hu X, Dong X, Cao F (2012). Surgical treatment of retrorectal tumors: a retrospective study of a ten-year experience in three institutions. Hepatogastroenterology.

[17] Koo YJ, Im KS, Jung HJ, Kwon YS (2012). Mature cystic teratoma of the uterosacral ligament successfully treated with laparoendoscopic single-site surgery. Taiwan J Obstet Gynecol.

[18] Afuwape OO, Ogundoyin OO, Ogunlana D, Adeleye A (2009). Adult sacrococcygeal teratoma: a case report. Ghana Med J.

[19] Monteiro M, Cunha TM, Catarino A, Tomé V (2002). Case report: sacrococcygeal teratoma with malignant transformation in and adult female: CT and MRI findings. Br J Radiol.

[20] Papadias K, Kairi-Vassilatou E, Kontogiani-Katsaros K, Argeitis J, Kondis-Pafitis A, Greatsas G (2005). Teratomas of the ovary: a clinico-pathological evaluation of 87 patients from one institution during a 10-year period. Eur J Gynaecol Oncol.

[21] Canelles E, Roig JV, Cantos M, García Armengol J, Barreiro E, Villalba FL, Ruiz MD, Pla V (2009). Presacral tumors. Analysis of 20 surgically treated patients. Cir Esp.

[22] Luk SY, Tsang YP, Chan TS, Lee TF, Leung KC (2011). Sacrococcygeal geratoma in adults: case report and literature review. Hong Kong Med J.

